# Chimeric RNA TNNI2-ACTA1-V1 Regulates Cell Proliferation by Regulating the Expression of NCOA3

**DOI:** 10.3389/fvets.2022.895190

**Published:** 2022-07-08

**Authors:** Dongyu Liu, Jiaxin Li, Wanjun Hao, Xu Lin, Jiqiao Xia, Jiyuan Zhu, Shuo Yang, Xiuqin Yang

**Affiliations:** College of Animal Sciences and Technology, Northeast Agricultural University, Harbin, China

**Keywords:** chimeric RNA *TA-V1*, *NCOA3*, porcine skeletal muscle, growth, cell proliferation

## Abstract

Chimeric RNA is a crucial target for tumor diagnosis and drug therapy, also having its unique biological role in normal tissues. *TNNI2-ACTA1-V1* (*TA-V1*), a chimeric RNA discovered by our laboratory in porcine muscle tissue, can inhibit the proliferation of Porcine Skeletal Muscle Satellite Cells (PSCs). The regulatory mechanism of *TA-V1* in PSCs remains unclear, but we speculate that *NCOA3, DDR2* and *RDX* may be the target genes of *TA-V1*. In this study, we explored the effects of *NCOA3, DDR2* and *RDX* on cell viability and cell proliferation by CCK-8 assay, EdU staining and flow cytometry. Furthermore, the regulatory pathway of proliferation in PSCs mediated by *TA-V1* through *NCOA3* or *CyclinD1* was elucidated by co-transfection and co-immunoprecipitation (Co-IP). The results revealed that overexpression of *NCOA3* significantly increased cell viability and the expression level of *CyclinD1*, and also promotes cell proliferation by changing cells from the G1 phase to the S phase. In addition, inhibiting the expression of *NCOA3* substantially reduced cell viability and inhibited cell proliferation. Overexpression of *DDR2* and *RDX* had no significant effect on cell viability and proliferation. Co-transfection experiments showed that *NCOA3* could rescue the proliferation inhibition of PSCs caused by *TA-V1*. Co-IP assay indicated that *TA-V1* directly interacts with *NCOA3*. Our study explores the hypothesis that *TA-V1* directly regulates *NCOA3*, indirectly regulating *CyclinD1*, thereby regulating PSCs proliferation. We provide new putative mechanisms of porcine skeletal muscle growth and lay the foundation for the study of chimeric RNA in normal tissues.

## Introduction

Chimeric RNAs are new RNAs formed by the fusion of two or more independent genes (1). The parental genes of chimeric RNAs can be located on different chromosomes, on different DNA strands of the same chromosome, or on the same DNA strand (2). In the past research, chimeric RNA has always been considered as a result of chromosomal rearrangement, a special marker for malignant tumors, and has attracted much attention on the diagnosis, treatment and prognosis of cancer (3). When they are found sporadically in normal samples, they are often considered “noise” from transcription, or as an artifact in reverse transcription and qPCR experiments (4). With the development of bioinformatics and the reduction of the cost of Next-generation sequencing, the development of chimeric RNA has been greatly promoted. With the deepening of research, more and more chimeric RNAs have been identified in normal tissues and cells (5). Some of these chimeric RNAs can play an important regulatory role in normal physiological activities (6). Therefore, the identification of chimeric RNA from more normal tissues will help us fully understand the composition and function of the genome.

Pigs are a crucial source of meat production worldwide and a potential medical model for human health issues (7). Muscle occupies a pivotal position in animal husbandry production. The quantity and quality of skeletal muscle are considered to be the main indicators for measuring meat quality (8). The growth and development of skeletal muscle is a very complex process, including the differentiation of muscle-derived stem cells into mononuclear myoblasts, fusion to form multinucleated myotubes, and mature muscle fibers (9). These processes involve the regulation of multiple myogenic factors. Therefore, research on the growth and development of skeletal muscle is great significance for improving the meat production and meat quality of livestock. Porcine Skeletal Muscle Satellite Cells (PSCs), as the progenitor cells of skeletal muscle, are considered to be the only source of stem cells for the myogenic differentiation of adult skeletal muscle cells (10). They are generally in a resting state. After being stimulated, skeletal muscle satellite cells can activate, proliferate and differentiate into muscle cells through the key signaling pathways, thereby participating in the formation and repair of skeletal muscle (11).

Chimeric RNA *TNNI2-ACTA1-V1* (*TA-V1*), one of the *TNNI2-ACTA1* transcript variants, is formed by trans-splicing of *TNNI2* and *ACTA1*. *TA-V1* was identified by our laboratory and our previous study found that *TA-V1* can inhibit the proliferation of PSCs (12). Interestingly, the parental genes do not have this function. *TA-V1* can inhibit the expression of *CyclinD1* and arrest the cell cycle in G1 phase, thereby inhibiting cell proliferation. Based on the transcriptomic analysis results, nuclear receptor coactivator 3 (*NCOA3*), discoidin domain receptor 2 (*DDR2*) and radixin (*RDX*) were specifically down-regulation. We speculate that one or more of these three genes may be involved in the process by which *TA-V1* inhibits the proliferation of PSCs.

In this study, we analyze the effect of candidate genes on cell proliferation by overexpression and siRNA. We explore the relationship between candidate genes and *TA-V1* through co-transfection and co-immunoprecipitation (Co-IP). The aim of this study is to reveal the molecular mechanism by which *TA-V1* regulates developmental in PSCs and to further explore the role of chimeric RNAs in normal tissues. These data considerably expanded the conclusions of our previous study and may shed new light on the developmental mechanisms of porcine skeletal muscle.

## Materials and Methods

### RNA Extraction and Reverse Transcription

According to the manufacturer's protocol, total RNA was isolated from Min pig longissimus dorsi or porcine skeletal muscle satellite cells using TRIzol reagent (Invitrogen, USA). Total RNA was reverse transcribed to cDNA using PrimeScript^TM^ RT reagent Kit (Takara, Japan). The purity and concentration of the RNA samples and cDNA samples were evaluated using a NanoDrop 2000 spectrophotometer (Thermo Scientific, USA).

### Expression Plasmid Constructions and siRNA

An eukaryotic expression vector (pCMV-HA) carrying the *NCOA3, DDR2* and *RDX* of pig was generated by cloning the coding sequence into the pCMV-HA vector. RNA samples from pig muscle were reverse-transcribed to cDNA, and full-length *NCOA3, DDR2* and *RDX* were amplified using primers in Supplementary Table 1. The eukaryotic expression plasmid pCMV-HA-TNNI2-ACTA1-V1 (pCMV-HA-TA-V1) was preserved by our laboratory. Both the short interfering RNA (siRNA) targeting the *NCOA3* coding region and the non-targeting negative control were designed and synthesized by Suzhou JiMa Company (Supplementary Table 2).

### Cell Culture and Transfection

PSCs were purchased from MingZhou Biological Technology Limited company (Ningbo, China). PSCs were cultured in DMEM/F-12 (Gibco, Shanghai, China) supplemented with 10% fetal bovine serum (FBS, CLARK, Shanghai, China) and 1% penicillin/streptomycin (Solarbio, Beijing, China). HEK293T cells were cultured in DMEM (Gibco, Shanghai, China) with 10% FBS and 1% penicillin/streptomycin. The cell culture medium was changed every 2 days. Cells were seeded in 6-well plates with growth medium. For overexpression, according to the manufacturer's instructions, cells at 70–80% confluent in serum-free DMEM/F12 or DMEM with gene expression vector or empty vector and Lipofectamine 2,000 reagent (Invitrogen, USA) for 6 h. For siRNA, the transfection procedure is similar to the overexpression transfection procedure. The experimental steps are strictly in accordance with the instructions (LipoRNAi^TM^ Transfection Reagent, Beyotime, Shanghai, China).

### CCK-8 Assay

The CCK-8 assay was used to detect cell viability, and the cells in each group were cultured and treated in the same conditions as above. These treated cells were plated in 96-well plates at 5,000 cells per well. At least three biological replicates were examined for each group. Next, 10 μL CCK-8 solution (Cell Counting Kit-8, Beyotime, Shanghai, China) was added in each well and the cell culture plate was incubated for 1 h. At last, the absorbance was read at 450 nm with a BIOTEK ELx800^TM^.

### EdU

According to the manufacturer's protocol, EdU staining was conducted using EdU kit (BeyoClick^TM^ EdU-488, Beyotime, Shanghai, China). Remove the medium from the treated cells and wash the cells carefully twice with PBS. Next, the cells were fixed in 4% paraformaldehyde for 30 min. The cells were incubated with EdU working solution for 30 min while protected from light. Hoechst 33,342 was used to stain the nucleus. The fluorescence intensity of each EdU-positive cell was quantified using Image J software.

### Cell Cycle

Cell cycle assays were performed at 48 h post-transfection. The cells were spun at 1,000 g for 5 min, and cell pellets were washed once in PBS at 4°C. Add 1 ml of 70% ethanol to each sample and incubate at 4°C for 2 h. The cells were spun at 1,000 g for 5 min, and cell pellets were washed once in PBS at 4°C. Remove the supernatant, add 1 mL propidium iodide (PI) working solution to each tube to re-suspend the pellets. Incubate for 30 min protected from light at 37°C. Cell cycle distribution was performed using an Accuri C6 flow cytometer (BD Biosciences). Data were analyzed using ModFit LT 5.0 (Verity Software House).

### Quantitative Real-Time-PCR Analysis

In order to detect the changes of *CyclinD1* and *NCOA3*, quantitative real-time-PCR analysis (qPCR) was performed (Supplementary Table 3). qPCR was performed using specific primers and SYBR Premix Ex Taq (Takara, Japan) on a BioRad iQ5 system (Bio-Rad, USA). Results were expressed by 2^−Δ*ΔCt*^ value and GAPDH was chosen as an internal reference.

### Western Blotting

Total proteins were prepared from cells using passive lysis buffer (Beyotime, Shanghai, China), and each sample was separated by sodium dodecyl sulfate polyacrylamide gel electrophoresis (SDS-PAGE) and transferred to polyvinylidene difluoride membranes. After blocking with tris-buffered saline with Tween 20 containing 5% skim milk, blots were incubated overnight at 4°C with primary antibodies. All membranes were then incubated with horseradish peroxidase-conjugated secondary antibodies (Abcam, UK) for 1 h at room temperature. Finally, the western blots were imaged using ChemiDoc XRS^+^(BIO-RAD, USA). The primary antibodies are as follows: Anti-HA taq (ab1424, Abcam, UK); Anti-NCOA3 (ab10310, Abcam, UK).

### Co-Immunoprecipitation

HEK 293T cells were transfected with plasmids 1 μg pCMV-HA-TA-V1 and 1 μg pCMV-HA-NCOA3. After 48 h, the cells were lysed on ice with 1 mL of cell lysis buffer (Beyotime, Shanghai, China) for 30 min. The cell lysates were then centrifuged at 12 000 rpm for 10 min. A total of 600 μL of the supernatants at a final concentration of 3 μg/μL were, respectively, precipitated with anti-NCOA3 monoclonal antibody, anti-HA monoclonal antibody in conjunction with Protein G Sepharose 4 Fast Flow (GE Healthcare Bio-Science) and were incubated with gentle rocking overnight at 4°C. The beads were washed five times with cold IP buffer and boiled with 5 × SDS loading buffer for 5 min. The immunoprecipitated proteins were detected by western blotting.

### Statistical Analysis

All statistical analyses were performed using GraphPad Prism 9.0 software (GraphPad Software, USA). A one-way analysis of variance and Student's *t*-test were used for data analysis and graph production. A probability (*P*) value of < 0.05 (^*^P < 0.05) was considered statistically significant and *P* < 0.01 (^**^P < 0.01) was considered to be highly significant.

## Results

### Overexpression of *NCOA3* Promoted PSCs Proliferation

**Figure 1:**
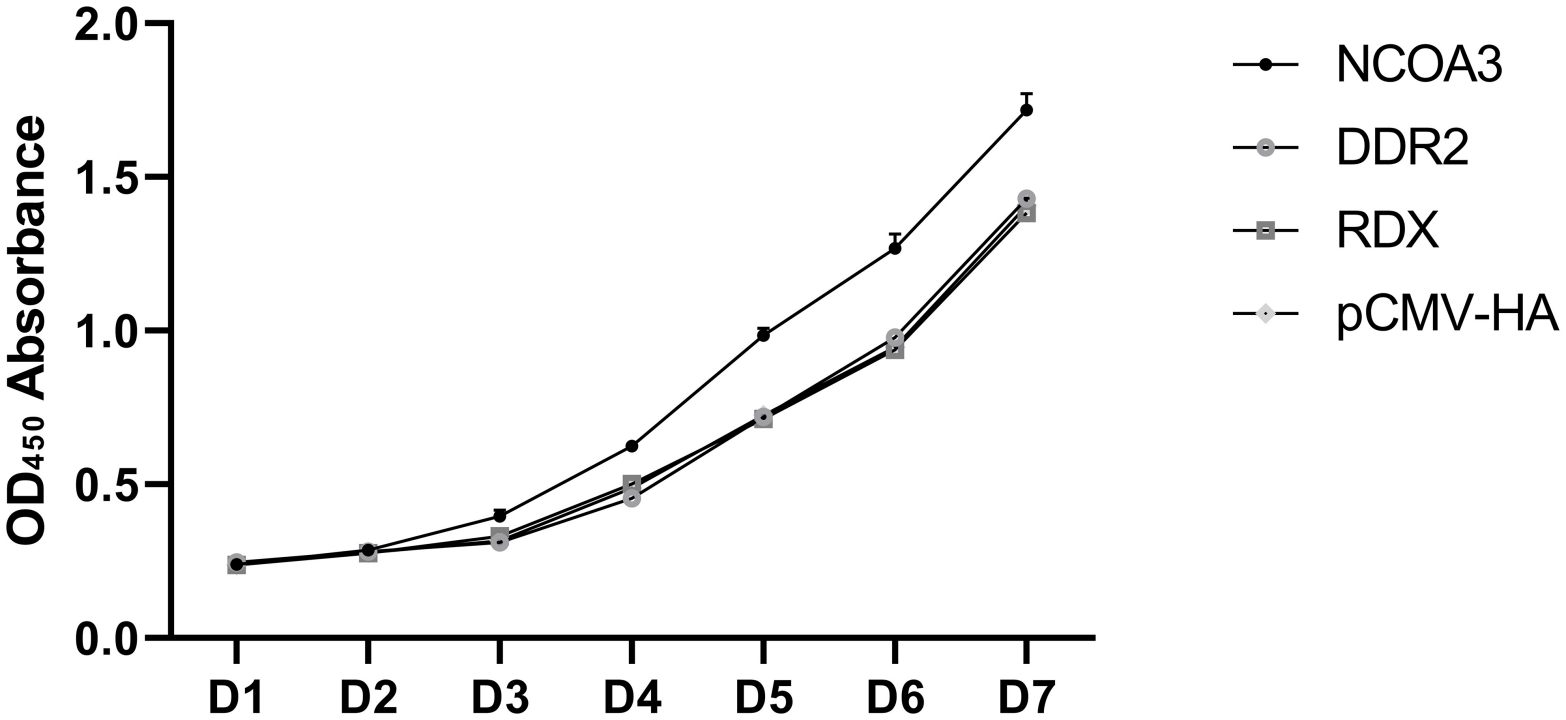
The results of the CCK-8 assay to detect cell viability in seven days.

**Figure 2:**
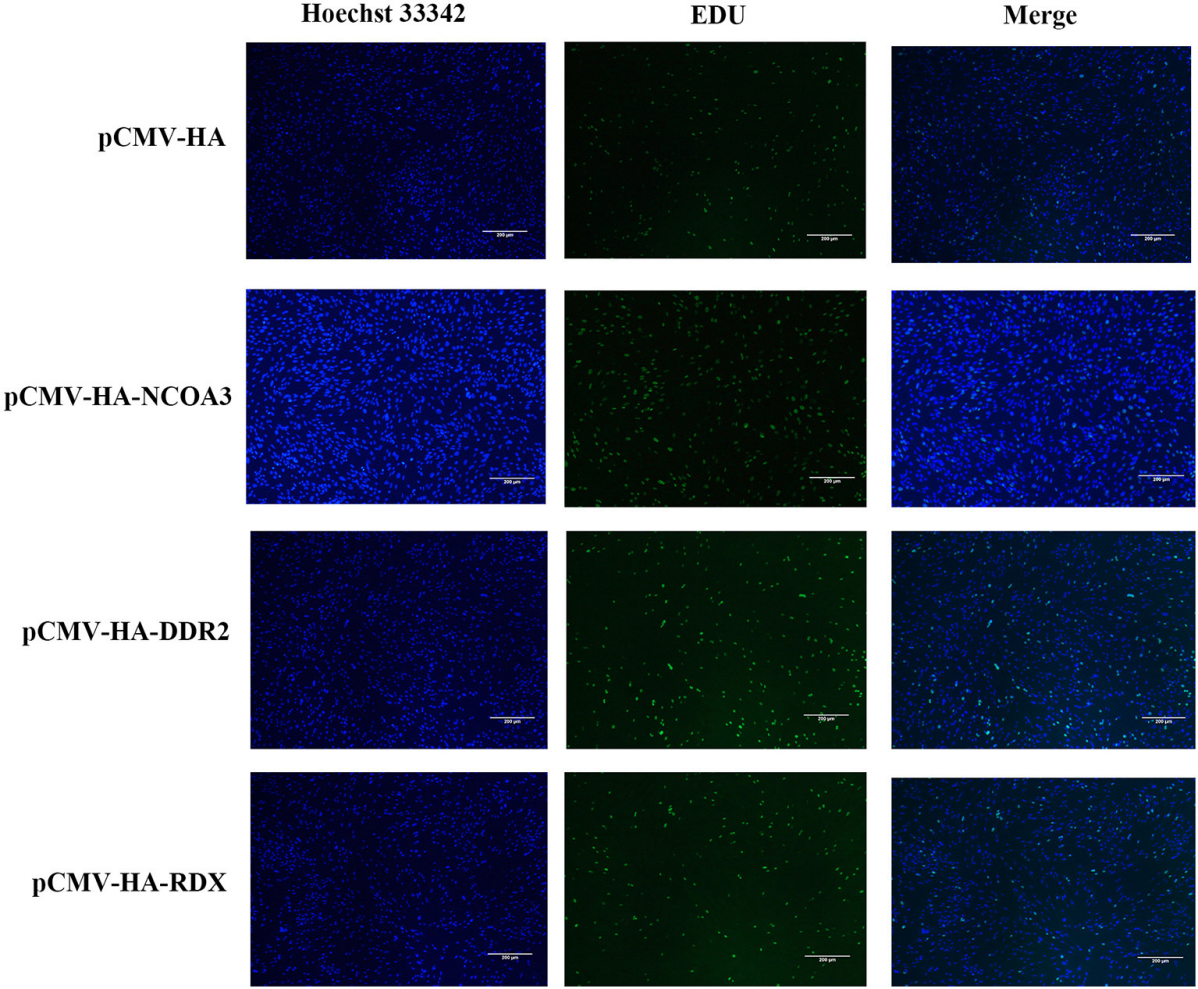
The effects of *NCOA3, DDR2* and *RDX* on cell proliferation of PSCs at 48 h post-transfection. EdU staining results of PSCs in pCMV-HA-NCOA3, pCMV-HA-DDR2 and pCMV-HA-RDX with magnification 100×.

**Figure 3:**
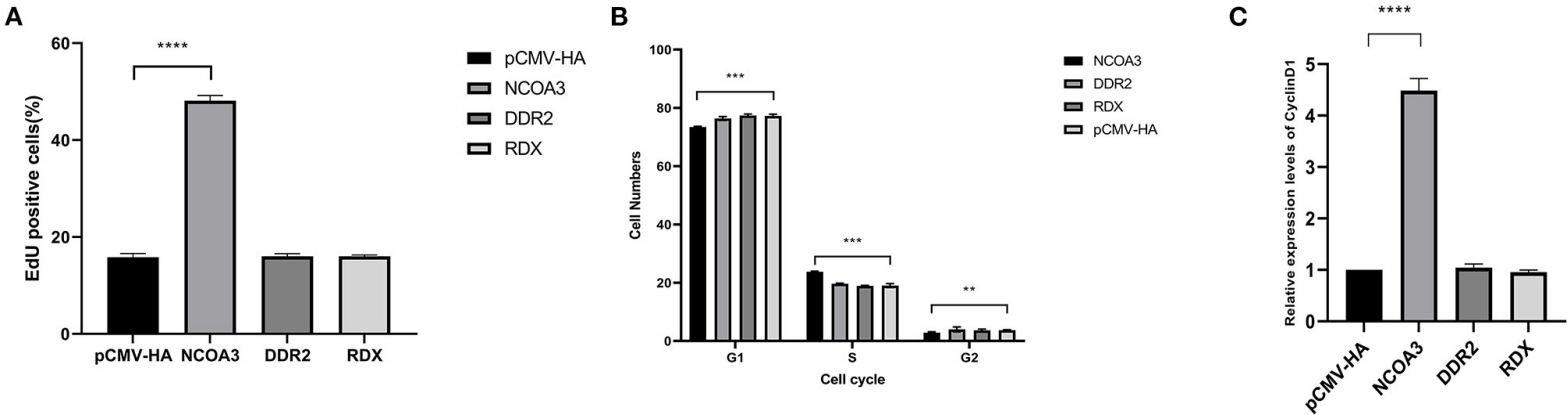
The effects of overexpression of target gene on EdU staining, cell cycle and expression level of *CyclinD1*. **(A)** The percentage of EdU-positive cells in *NCOA3, DDR2* and *RDX* groups. **(B)** Effect of target gene on cell cycle. Cell samples from each group were collected at 48 h post-transfection. **(C)** Detection of mRNA expression level of *CyclinD1* by qPCR. A value of *p* < 0.05 was considered statistically significant. **p < 0.01, ***p < 0.001, ****p < 0.0001.

To investigate the regulatory function of *NCOA3, DDR2* and *RDX* on PSCs proliferation, an overexpression experiment was performed by transfecting aeukaryotic expression vector into PSCs. The CCK-8 assay was used to detect the effect of transfection of plasmid vector on PSC viability. The CCK-8 assay results revealed that the PSCs viability was significantly higher in the *NCOA3* group than that in the NC group (Figure 1) (Supplementary Table 4). We then analyzed the cell proliferation by EdU staining. The nuclei of all PSCs were stained with blue (Hoechst 33342), and the nuclei of PSCs with high DNA replication activities (EdU-positive cells) were stained with green simultaneously. The results showed significantly increased EdU-positive cells after transfection *NCOA3*, compared with the control (Figure 2). EdU assay results showed that the number of proliferating PSCs in the *NCOA3* group was increased (*P* < 0.0001), compared with the NC group (Figure 3A). There were no significant differences between the *DDR2* and *RDX* groups in CCK-8 assay and EdU staining compared to the NC group.

Further, we examined the cell cycle after overexpression of eukaryotic plasmids. After transfection with plasmid pCMV-HA-NCOA3 for 48h, the proportion of PSCs in G1 phase the most significantly decreased from 77.25 ± 0.47% to 73.41 ± 0.22% (*P* < 0.001); the proportion in S phase extremely significant increased from 19.02 ± 0.55% to 23.76 ± 0.15% (*P* < 0.001); the proportion in G2 phase significantly decreased from 3.73 ± 0.1% to 2.83 ± 0.25% (*P* < 0.01) (Figure 3B). In order to verify whether *NCOA3* is involved in the process of *TA-V1* regulating cell proliferation, we detected the expression level of *CyclinD1* by qPCR. The results show that the expression level of *CyclinD1* was significantly increased in the *NCOA3* group (*P* < 0.01) (Figure 3C). Overexpression of *DDR2* and *RDX* had no significant effect on cell cycle and mRNA expression levels of related genes.

### Inhibition of *NCOA3* Inhibited the Proliferation of PSCs

**Figure 4:**
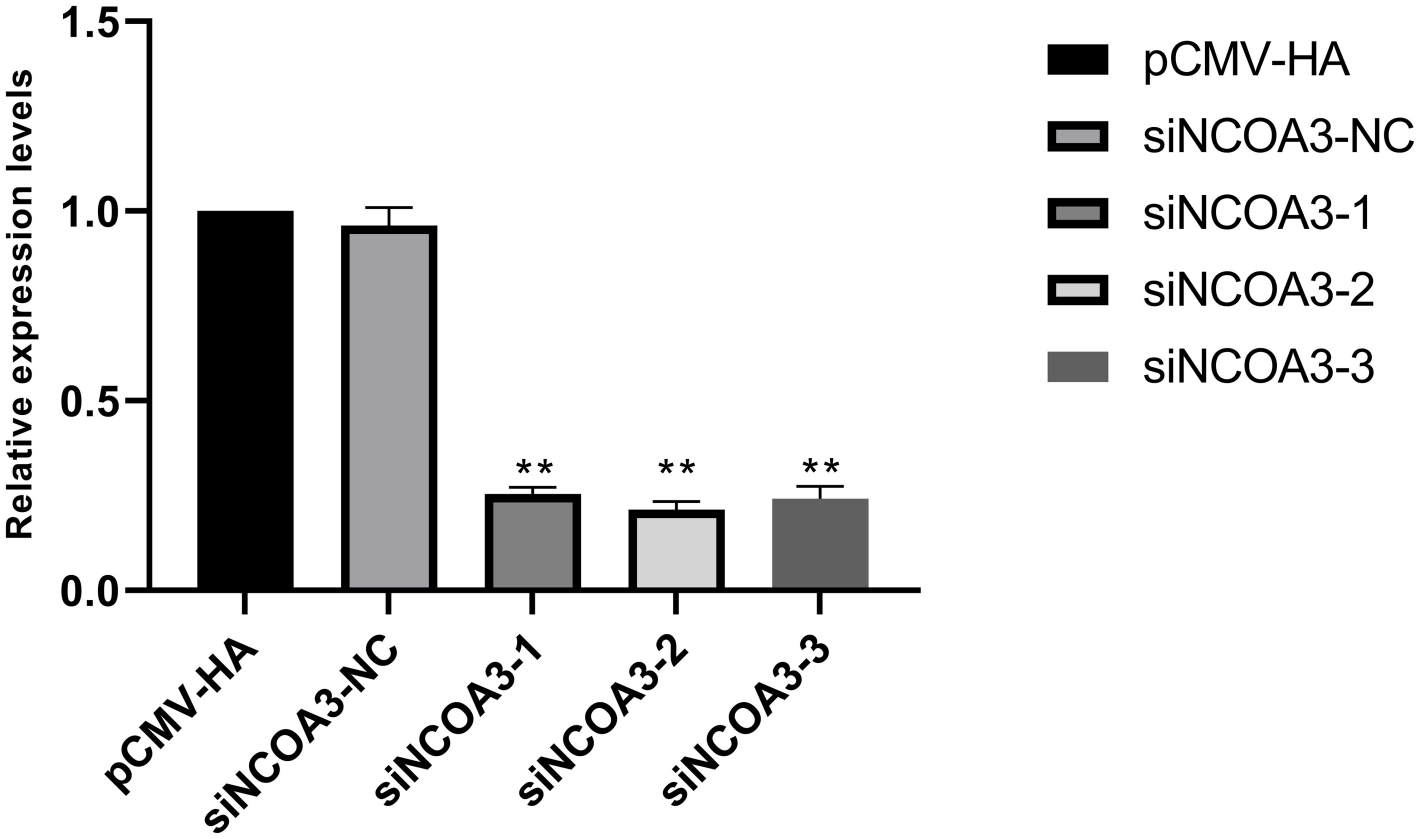
The results of siRNA screening, the lower the expression level of *NCOA3*, the better the inhibitory effect. **p < 0.01.

**Figure 5:**
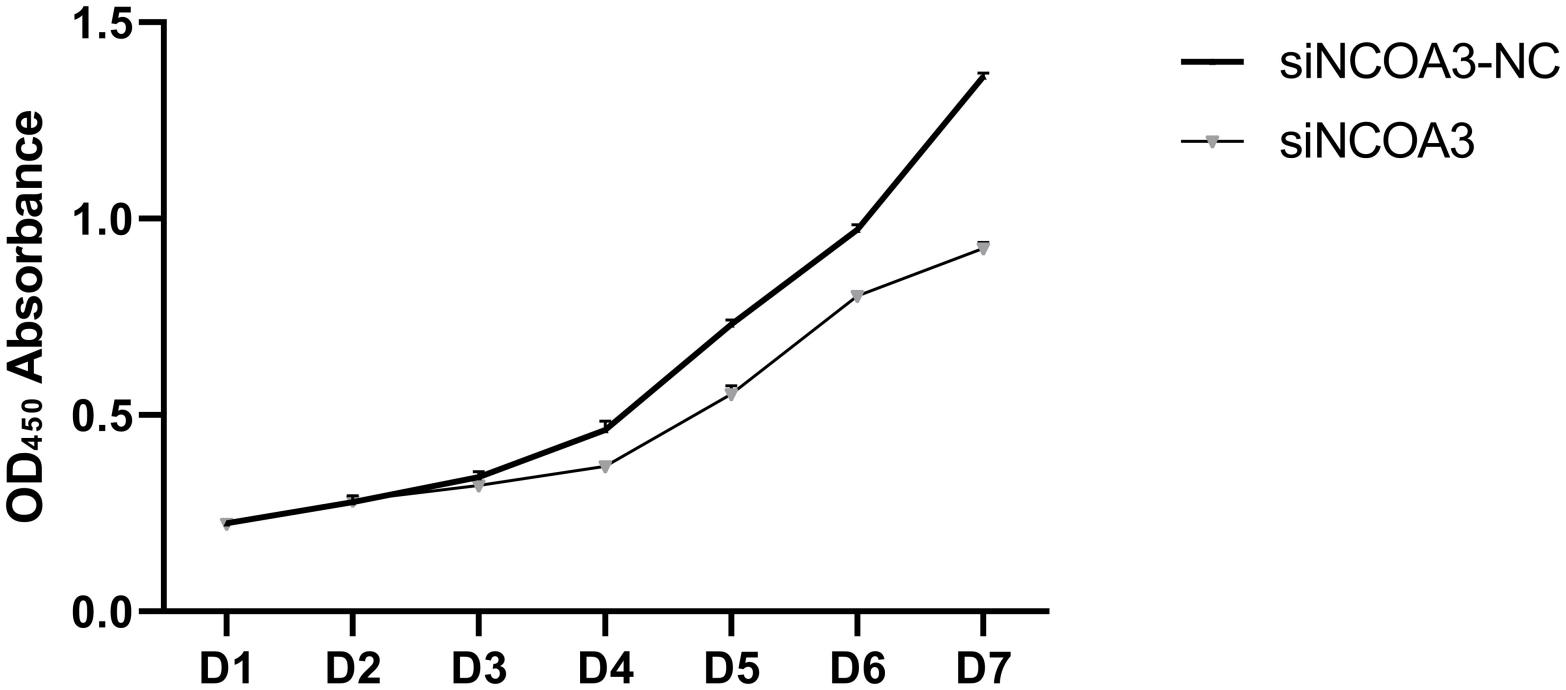
The effect of interfering expression of *NCOA3* on cell viability of PSCs. The assay lasts for seven days.

**Figure 6:**
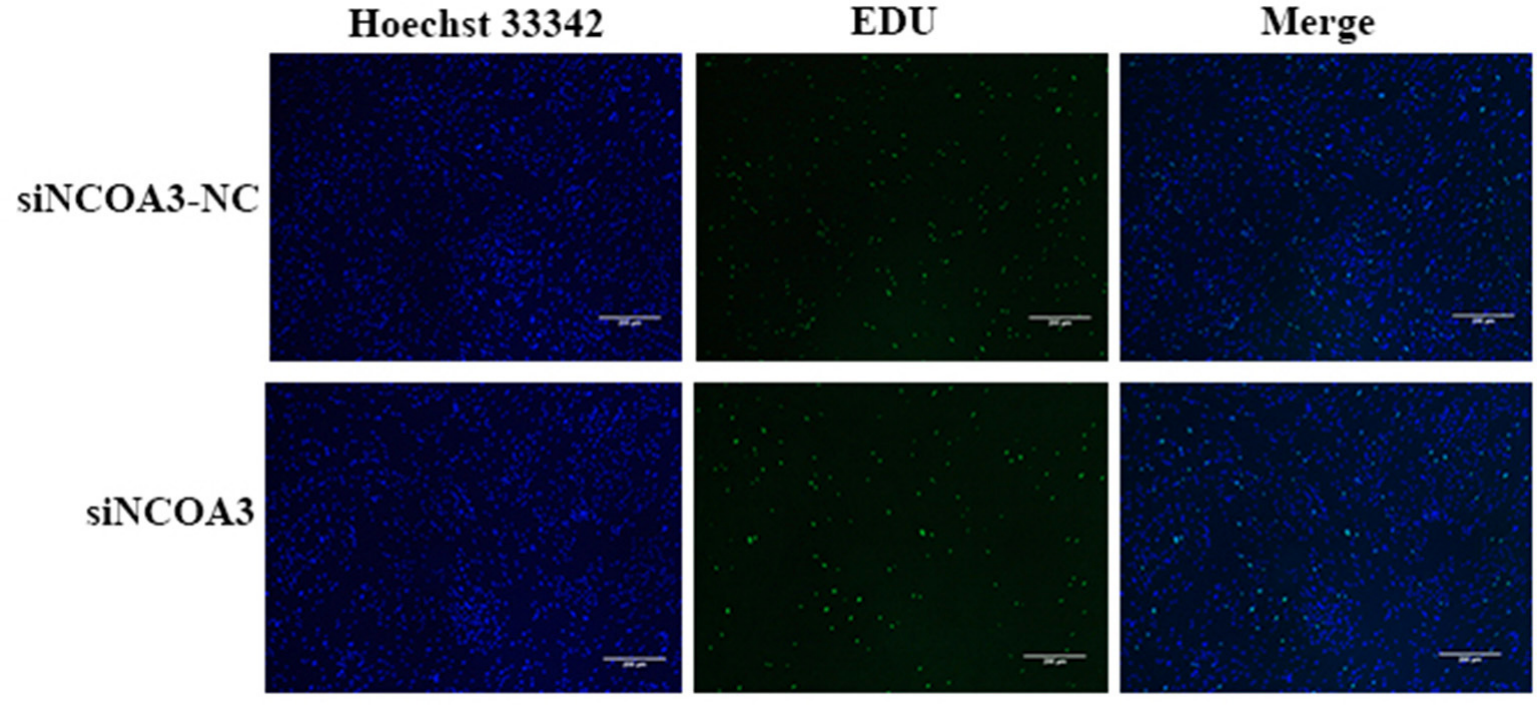
The effect of interfering expression of *NCOA3* on the proliferation of PSCs. The effect of inhibiting the expression of *NCOA3* on EdU staining. The results of the percentage of EdU positive cells after interfering expression of *NCOA3*.

**Figure 7:**
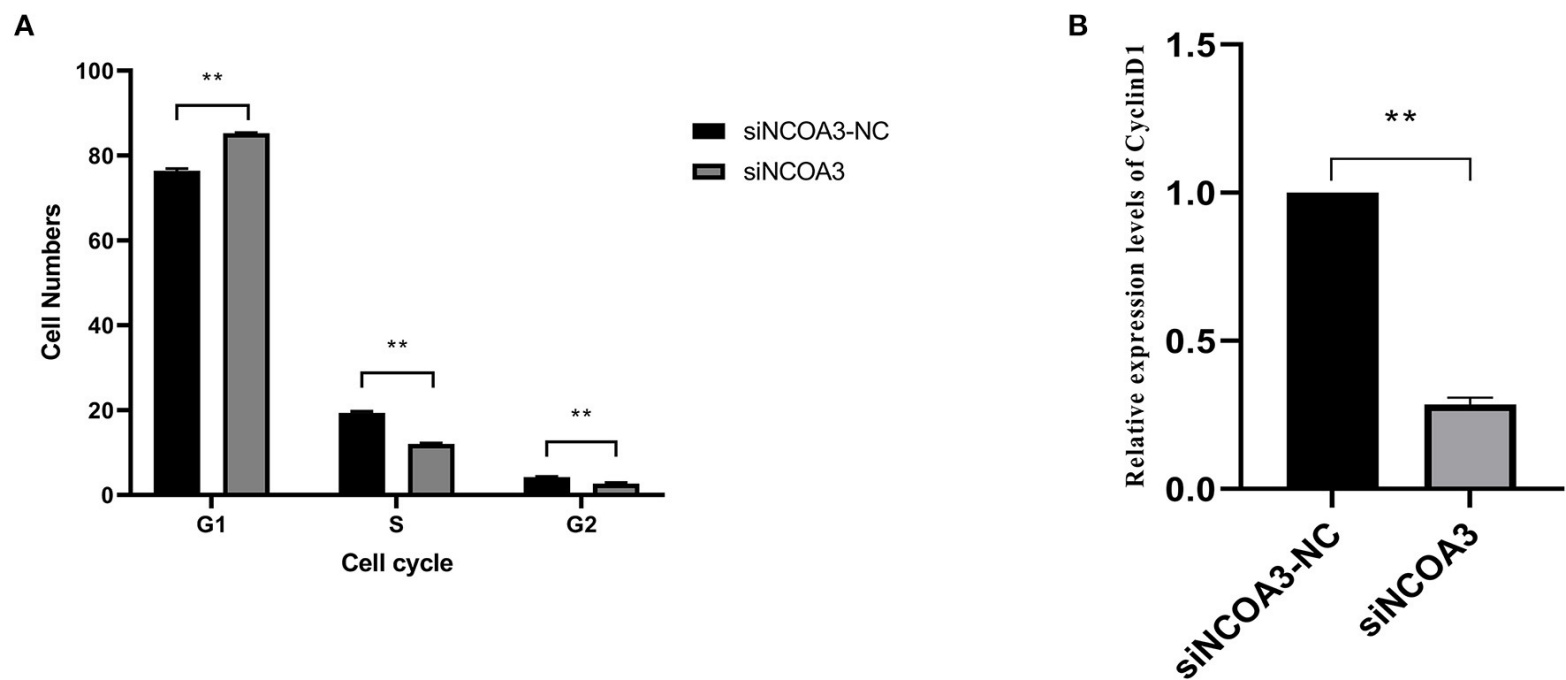
The effects of inhibiting *NCOA3* expression on cell cycle and expression level of *CyclinD1*. **(A)** The effects on cell cycle after interfering expression of *NCOA3*. **(B)** After 48 h of interference expression of *NCOA3*, cells were collected. The expression levels of *CyclinD1* were detected. **p < 0.01.

To further explore the role of *NCOA3* in PSCs, siRNA was transfected into PSCs. First, we screened for siRNAs by qPCR. We chose siNCOA3-2 for subsequent experiments and named it siNCOA3 (Figure 4). The CCK-8 assay and EdU staining revealed that the PSCs proliferation in the siNCOA3 group was significantly lower than that of the NC group (Figures 5, 6) (Supplementary Table 5). After interfering with *NCOA3* expression, the proportion in G1 phase increased significantly, and the proportion in S phase and G2 phase decreased significantly (Figure 7A). The expression level of *CyclinD1*, a key gene in G1-S phase, was significantly down-regulated after siNCOA3 transfection (Figure 7B) (*P* < 0.01). These results showed that the inhibition of *NCOA3* could inhibit the proliferation of PSCs. From the above results, we demonstrate that *NCOA3* is essential during the proliferation of PSCs.

### *TA-V1* Regulates the Expression Level of *NCOA3* and *CyclinD1*

**Figure 8:**
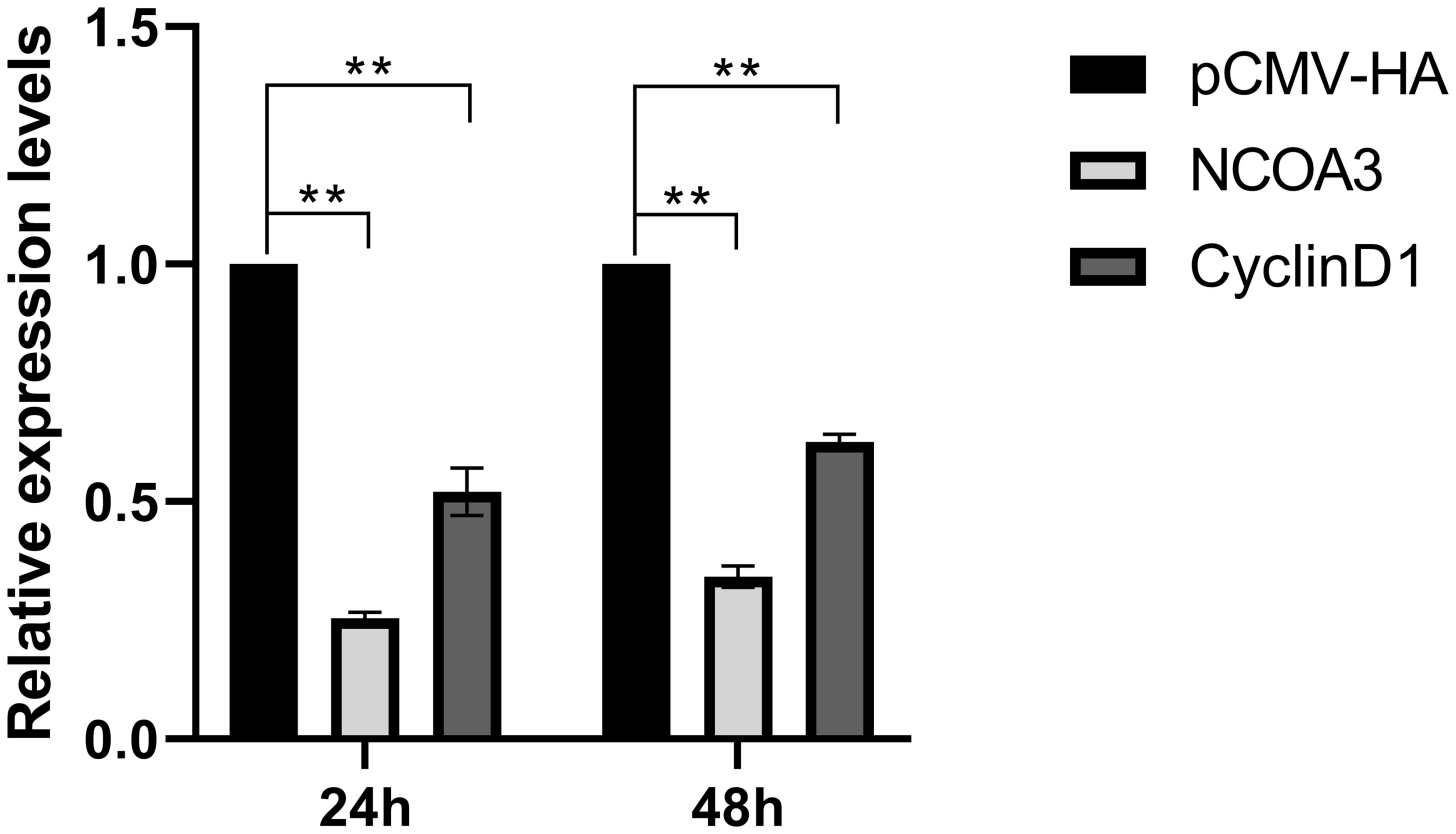
At post-transfection of pCMV-TA-V1, cells were collected at 24 and 48 h, respectively. The expression levels of *NCOA3* and *CyclinD1* were detected, respectively, by qPCR. **p < 0.01.

To further explore the role of *TA-V1* in PSCs, we examined the mRNA expression levels of *NCOA3* and *CyclinD1* after overexpression of *TA-V1* at 24 h and 48 h. The expression levels of *NCOA3* and *CyclinD1* significantly decreased after overexpression of *TA-V1* (Figure 8) (*P* < 0.01). This result is consistent with the transcriptomic analysis results. Based on our lab previous experimental results, we have reasons to speculate that *TA-V1* regulates the proliferation of PSCs by regulating *NCOA3*.

### Overexpression of *NCOA3* Can Counteract the Proliferation Inhibition Caused by Overexpression of *TA-V1*

**Figure 9:**
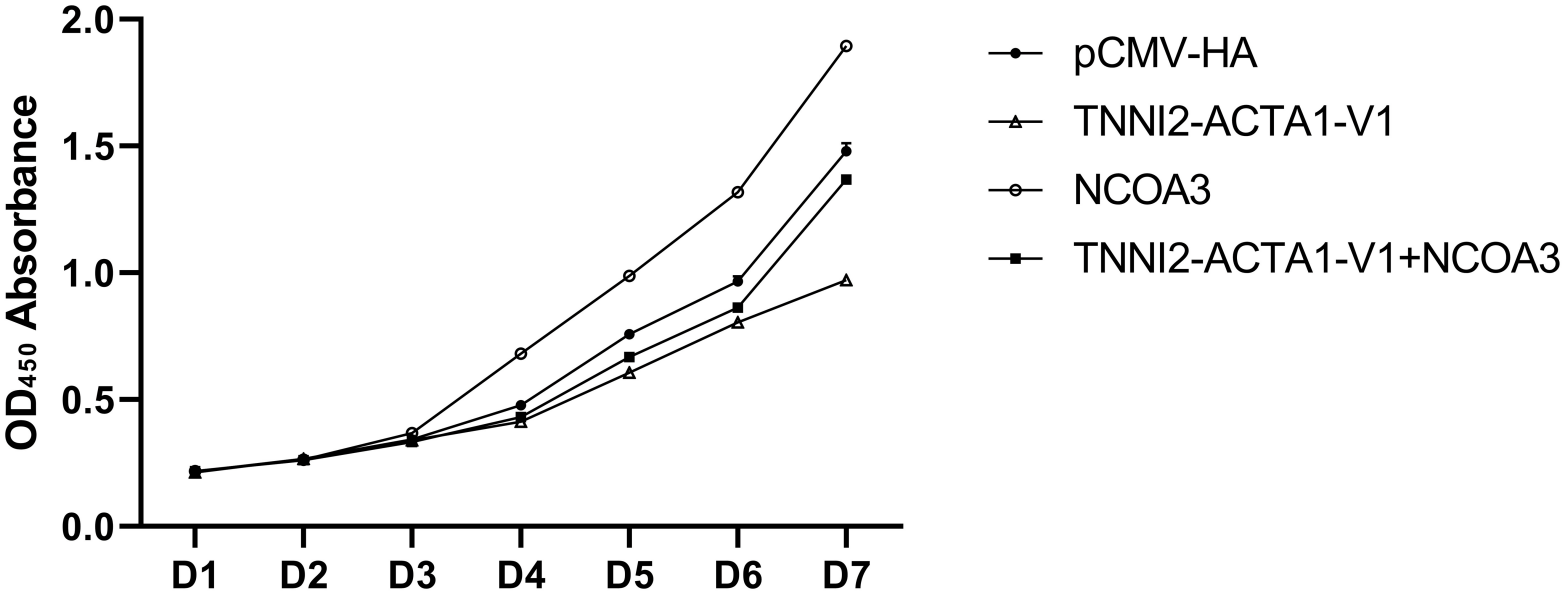
The effects of co-transfection of *TA-V1* and *NCOA3* on cell viability.

**Figure 10:**
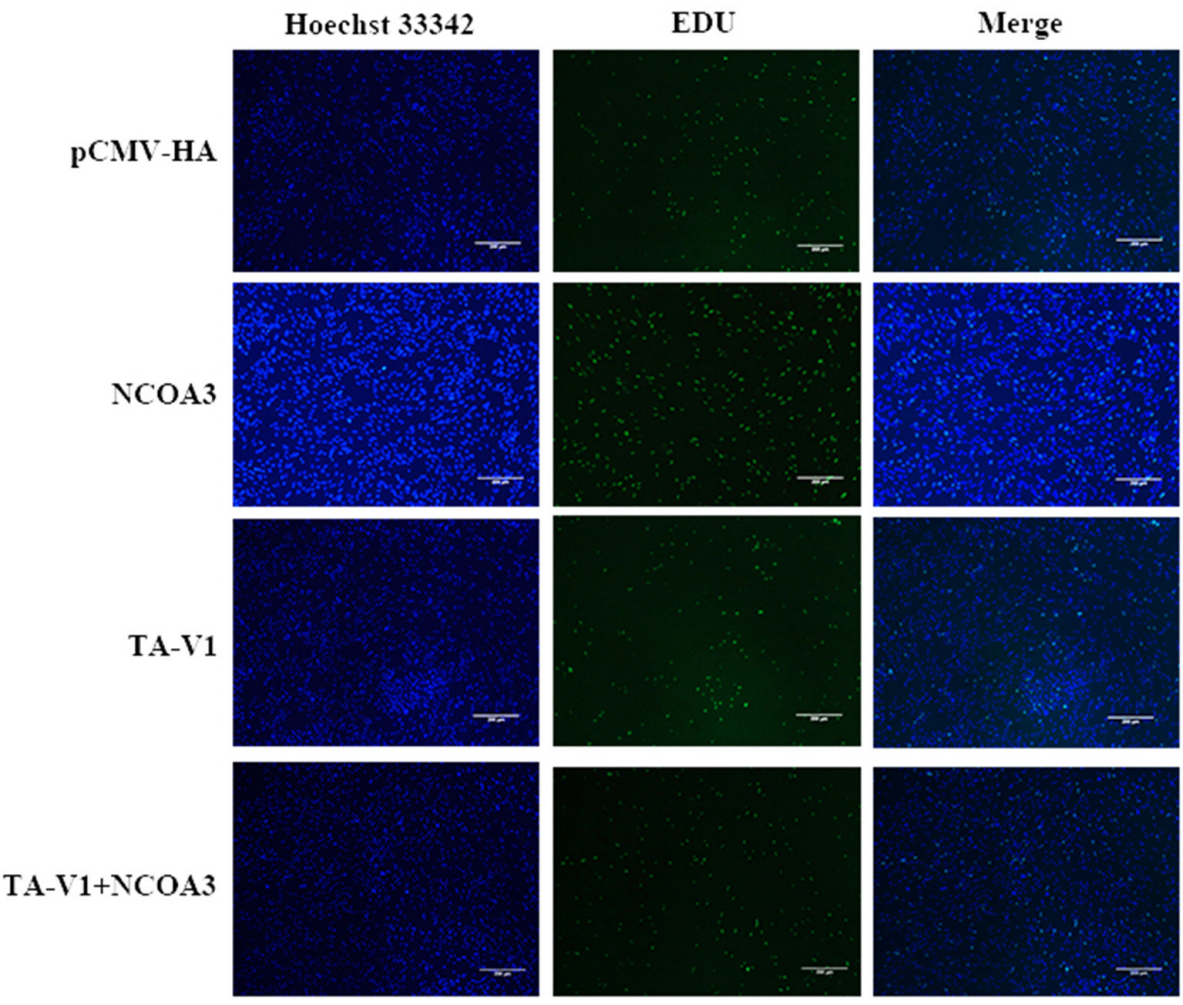
The effects of co-transfection of *TA-V1* and *NCOA3* on cell proliferation. The effect of co-transfection of *TA-V1* and *NCOA3* on EdU staining is shown.

**Figure 11:**
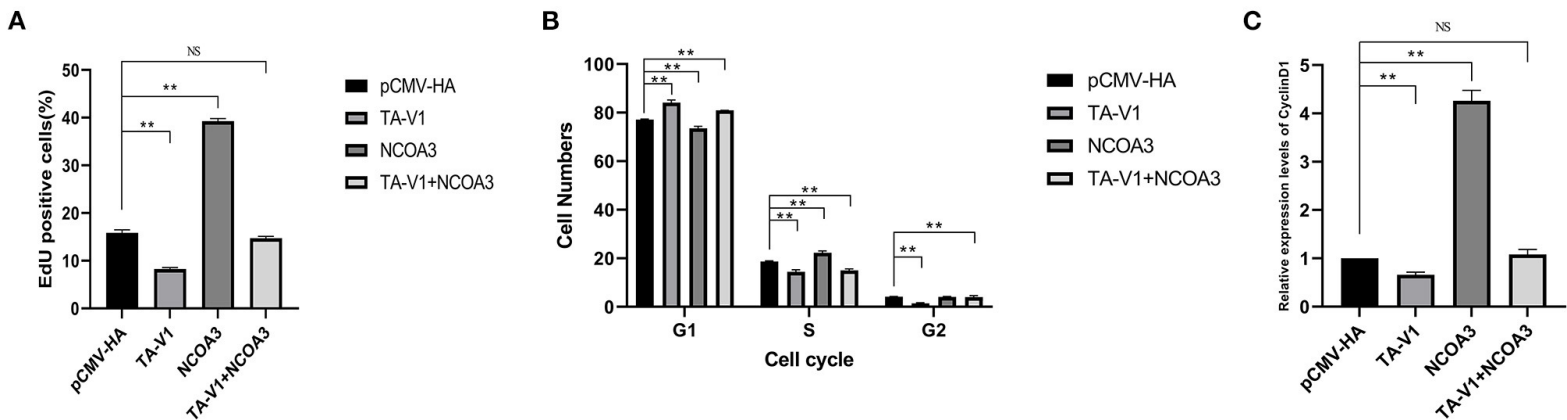
The effects of co-transfection of *TA-V1* and *NCOA3* on EdU staining, cell cycle and expression level of *CyclinD1*. **(A)** The percentage of EdU positive cells after co-transfection of *TA-V1* and *NCOA3*. **(B)** The effects on cell cycle after co-transfection of *TA-V1* and *NCOA3*. **(C)** The effects of co-transfection of *TA-V1* and *NCOA3* on the expression level of *CyclinD1*. The NS means no significant. **p < 0.01.

To further verify our speculation, we co-transfected pCMV-HA-TA-V1 and pCMV-HA-NCOA3 into PSCs to verify whether *NCOA3* has the ability to rescue the proliferation inhibition caused by *TA-V1*. The CCK-8 assay results showed that the cell viability in the *TA-V1* group was significantly decreased, and the cell viability in the *NCOA3* group was significantly increased. Although the cell viability in the co-transfection group was lower than the NC group, it was significantly higher than *TA-V1* group (Figure 9) (Supplementary Table 6). The results of EdU staining demonstrated that the *TA-V1* group significantly inhibited the proliferation of PSCs, and the *NCOA3* group significantly promoted the proliferation of PSCs (Figures 10, 11A). There was no significant difference between the co-transfection group and the NC group.

The cell cycle results showed that the G1 phase of *TA-V1* group, *NCOA3* group and co-transfection group were 84.12 ± 0.89%, 73.58 ± 0.67% and 80.95 ± 0.04 %, respectively; the S phase were 14.46 ± 0.66%, 22.31 ± 0.61% and 15.03 ± 0.48%, respectively; G2 phase were 1.42 ± 0.23%, 4.11 ± 0.16% and 4.02 ± 0.52%, respectively (Figure 11B). Overexpression of *TA-V1* can arrest the cell cycle in G1 phase, and overexpression of *NCOA3* can promote cells from G1 phase to S phase. Although the number of cells in G1 phase in the co-transfection group was more than that in the *NCOA3* group, it was less than that in the *TA-V1* group. The results of qPCR showed that the expression level of *CyclinD1* in *TA-V1* group was significantly down-regulated, and the expression level of *CyclinD1* in *NCOA3* group was significantly up-regulated (Figure 11). Compared with the NC group, there was no significant difference in the expression level of *CyclinD1* in the co-transfection group. These findings indicated that overexpression of *NCOA3* can counteract the proliferation inhibition caused by overexpression of *TA-V1*.

### Direct Binding of *TA-V1* and *NCOA3*

**Figure 12:**
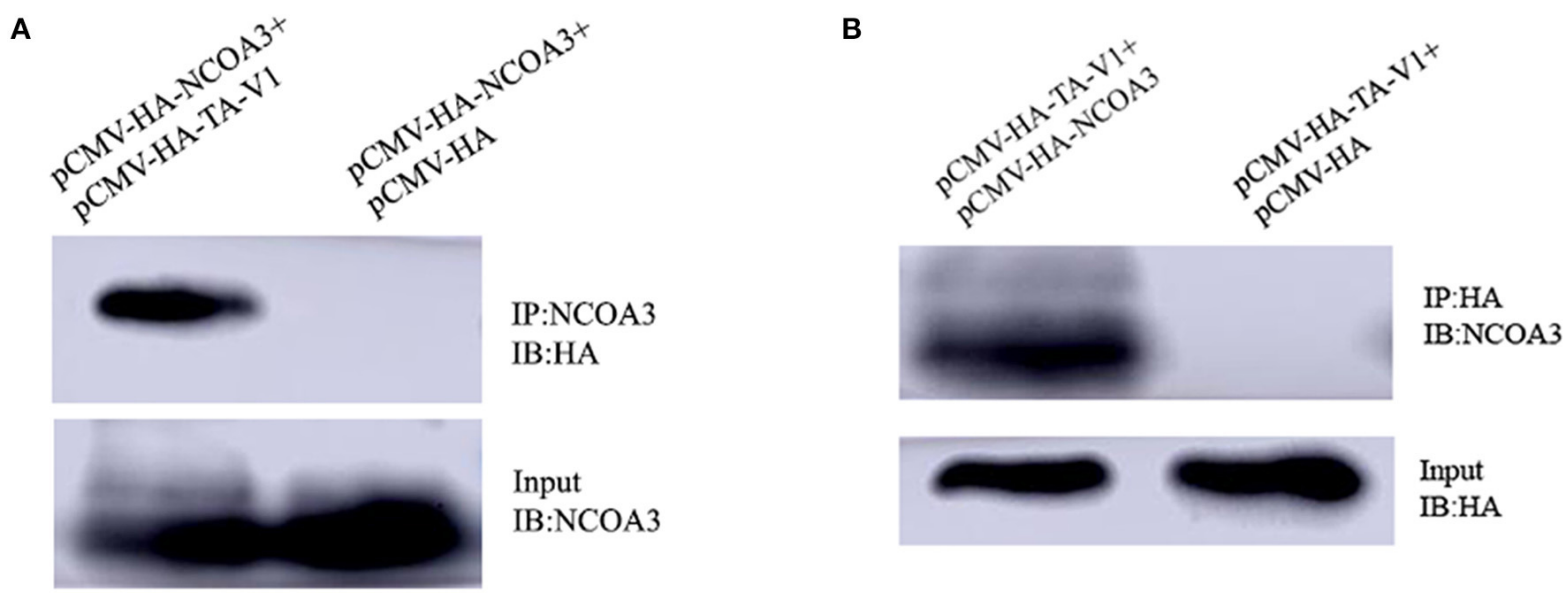
Identification of the protein-protein interaction between *TA-V1* and *NCOA3* using Co-immunoprecipitation (Co-IP). HEK293T cells were transfected with pCMV-HA-TA-V1, pCMV-HA-NCOA3 or empty vector pCMV-HA. Cells were treated as described in Materials and Methods. **(A)** Anti-NCOA3 antibody was used as the primary antibody for Co-IP. **(B)** Anti-HA antibody was used as the primary antibody for Co-IP.

We verified whether *TA-V1* could interact with the *NCOA3* protein by immunoprecipitation. In HEK 293T cells, when pCMV-HA-TA-V1 was co-transfected with pCMV-HA-NCOA3, the results showed that HA-TA-V1 protein was detected after the anti-NCOA3 antibody was used to immunoprecipitate *NCOA3*. And there was no band when pCMV-HA was transfected as the control vector. Then, we discovered that *NCOA3* could be detected after using an anti-HA antibody to immunoprecipitate *TA-V1*. Co-IP experiments indicate that *TA-V1* can directly interact with *NCOA3* (Figure 12).

## Discussion

Chimeric RNAs are new RNAs formed by the fusion of two or more independent genes. For the past several decades, chimeric RNAs are thought to be the product of chromosomal rearrangements (13, 14). With the deepening of research, it has been found that chimeric RNAs can be formed by different splicing, including cis-splicing of adjacent genes (cis-SAGe) and trans-splicing (trans-splicing, TS) (15–18). Chimeric RNAs and their encoded products are considered unique features of cancer. With the advancement of medical technology, some of them are used as diagnostic markers and drug targets (19). However, recent studies have revealed the existence of chimeric RNAs in non-cancer tissues (20). Studies have found that chimeric RNAs are not only related to cancer, but also to normal physiological functions. Some chimeric RNAs are involved in life processes such as cell proliferation, apoptosis, cycle regulation, and maintenance of functional diversity (21).

Chimeric RNA *TA-V1* was analyzed and identified from RNA-Seq data sets of normal muscle tissue. It was formed by the fusion of the first six exons of *TNNI2* and the last four exons of *ACTA1*. Our previous experimental results showed that overexpression of *TA-V1* inhibited the expression of *CyclinD1*, resulting in the arrest of the G1 phase, thereby inhibiting the proliferation of PSCs. Interestingly, the parental gene and other transcript variants do not have this function. To further explore the role of *TA-V1* on cell proliferation, next-generation sequencing was used to perform transcriptome analysis. The results showed that three genes (*NCOA3, DDR2* and *RDX*) were specifically enriched compared to the parental genes (12).

In order to explore *TA-V1* inhibits the proliferation of PSCs potential mechanisms, we focused on the effects of *NCOA3, DDR2* and *RDX* on the proliferation of PSCs. In this study, we designed and constructs the plasmids, pCMV-HA-NCO3A, pCMV-HA-DDR2 and pCMV-HA-RDX, to enhance these mRNA expression levels in PSCs. The CCK-8 assay and EdU staining results showed that *NCOA3* promotes the proliferation of PSCs. Conversely, down-regulation of *NCOA3* expression inhibited the proliferation of PSCs. These results suggest that *NCOA3* plays a notable role in the proliferation of PSCs. However, overexpression of *DDR2* and *RDX* did not significantly effect the proliferation of PSCs.

The most important thing in the growth process of the body is the synthesis of new cells. These new cells are formed through the division of mother cells. In a nutshell, the mother cell replicates its own material and then divides to form two daughter cells. The newly divided cell also becomes the mother cell, starting a new cycle of synthesis and division. We define the above cell replication and division process as the cell cycle (4). In the reproduction of eukaryotes, the cell cycle plays an extremely important role and runs through the entire life process of the organism (22). Consequently, the orderly operation of the cell cycle is strictly regulated by a variety of genes. For instance, *Cyclins*, cyclin-dependent kinases (*CDKs*), and CDK inhibitors (*CKI*) (23). After overexpression of *NCOA3* in PSCs, the proportion of G1 phase and G2 phase cells is decreased (*P* < 0.01), and the proportion of S phase cells is increased (*P* < 0.01). This result indicates that *NCOA3* can promote the cell cycle G1 phase into S phase, thereby shortening the time from G1 phase to S phase. The G1-S phase is the first molecular switch in the cell cycle that controls cells to continue dividing or enter a quiescent state (24). Numerous studies have shown that efficient expression of *CyclinDs* is required for entering S phase from G1 phase (25, 26). To verify whether *NCOA3* is involved in the regulation of *CyclinD1* by *TA-V1*, qPCR was performed. We detected the expression level of *CyclinD1* and found that the expression level of *CyclinD1* was increased after overexpression of *NCOA3*. Conversely, the expression level of *CyclinD1* was significantly decreased after inhibiting *NCOA3*. In addition, overexpression of *DDR2* and *RDX* did not significantly affect the cell cycle of PSCs. Based on the above results, we concluded that *NCOA3* can regulate the cell cycle by regulating the expression level of *CyclinD1*, thereby regulating the proliferation of PSCs. According to this result, we have reason to speculate that *TA-V1* regulates the proliferation of PSCs by regulating the expression level of *NCOA3*.

Nuclear receptor coactivator 3 (*NCOA3*), also known as Steroid receptor coactivator 3 (*SRC3*) or Amplified in breast cancer 1 (*AIB1*), is a member of the p160 steroid receptor coactivator family (27). Recent research has shown that *NCOA3* plays an important role in the process of gene expression and regulation by combining with the nuclear receptor. *NCOA3* can interact with nuclear receptors such as estrogen receptor and androgen receptor (*AR*) and other transcription factors such as activator protein-1 (*AP-1*), nuclear factor-kB (*NF-kB*) and *E2F1* to enhance its action about target gene transcription (28, 29). Chen's research showed that knocking out *NCOA3* in mice caused developmental delays (30). These results suggest that *NCOA3* may be required for cell growth. Some scholars suggest that *NCOA3* is involved in estrogen-dependent transcriptional regulation of *CyclinD1* (31). Karmakar found that knockdown of *NCOA3* could reduce 17β-estradiol-induced expression level of *CyclinD1* to some extent (32). Zhou's study shows that *NCOA3* can interact with *E2F1* to promote the proliferation of hormone-dependent and independent breast cancer cells (33). Some scholars reported that cell proliferation was inhibited and the cell cycle G1/S phase was arrested by down-regulating the expression of *NCOA3* in prostate cancer cells (27). These findings suggest that *NCOA3* plays a significant role in cell growth and development.

In order to verify our conjecture, we performed co-transfection experiments. The CCK-8 assay results showed that the cell viability in the co-transfection group was significantly higher than the *TA-V1* group and slightly lower than the NC group. The EdU staining results showed that there was no significant difference in the EdU positive cells between co-transfection group and the NC group. The expression level of *CyclinD1* was not significantly different between the co-transfection group and the NC group. Co-transfection experiments result showed that overexpression of *NCOA3* could rescue the proliferation inhibition of PSCs caused by overexpression of *TA-V1*. In order to deeply explore the relationship between *TA-V1* and *NCOA3*, we designed a Co-IP experiment which showed that *TA-V1* protein can directly bind to *NCOA3* protein.

In summary, the CCK-8 assay and EdU staining results showed that overexpression of *NCOA3* can promote the proliferation of PSCs, while the other two candidate genes (*DDR2* and *RDX*) had no significant effect on the proliferation of PSCs. The number of cells in G1 phase was significantly decreased and the expression level of *CyclinD1* was significantly increased by transfection pCMV-HA-NCOA3. Conversely, inhibiting the expression level of *NCOA3* inhibited the proliferation of PSCs. After transfection siNCOA3, PSCs were arrested in the G1 phase and the expression level of *CyclinD1* was decreased. After overexpression of *TA-V1*, the expression levels of *NCOA3* and *CyclinD1* were significantly decreased. The results of co-transfection experiments showed that *NCOA3* rescues *TA-V1*-induced proliferation inhibition of PSCs. Co-ip experiments indicated that *TA-V1* directly binds to *NCOA3*, thereby regulating cell proliferation. The above results indicate that *TA-V1* directly regulates the expression level of *NCOA3* and thus indirectly regulates the expression level of *CyclinD1*, thereby regulates the proliferation of PSCs. In short, our research explores the mechanism of *TA-V1* effects the proliferation of PSCs, providing new ideas for the study of muscle growth. At the same time, our research also provides a theoretical basis for the role of chimeric RNAs in normal tissues.

## Data Availability Statement

The raw data supporting the conclusions of this article will be made available by the authors, without undue reservation.

## Author Contributions

DL and XY came up with and designed the study. DL performed the experiments and wrote the manuscript. JL, WH, XL, JX, JZ, and SY contributed reagents, materials, and analysis tools. XY revised the manuscript. All authors contributed to the article and approved the submitted version.

## Funding

This research was supported by the National Natural Science Foundation of China (32172696).

## Conflict of Interest

The authors declare that the research was conducted in the absence of any commercial or financial relationships that could be construed as a potential conflict of interest.

## Publisher's Note

All claims expressed in this article are solely those of the authors and do not necessarily represent those of their affiliated organizations, or those of the publisher, the editors and the reviewers. Any product that may be evaluated in this article, or claim that may be made by its manufacturer, is not guaranteed or endorsed by the publisher.
