# Supplementary material for: Chimeric RNA TNNI2-ACTA1-V1 Regulates Cell Proliferation by Regulating the Expression of NCOA3
Source: Front Vet Sci. 2022 Jul 8;9:895190. doi: 10.3389/fvets.2022.895190 (PMC9309209; doi:10.3389/fvets.2022.895190)
Supplement: Supplementary file 1 [file Data_Sheet_1.PDF]

Supplementary Material

1 Supplementary Tables

1.1 Primer sequences for expression plasmid constructions were shown in Supplementary Table 1.

Supplementary Table 1. Primer sequences for expression plasmid constructions

Gene	Gene bank	Primer sequences（5’-3’）	Production length(bp)
NCOA3	NM_001114276.1	F:GAATTCCGATGATGAGTGGATTAG<br>R:GGTACCCTCAGCAATATTTCTGATCAGG	4173
DDR2	XM_013988083.2	F:GAATTCCGATGGGGACGATTGCTGGAC<br>R:GGTACCCTCACTCGTCCCCTTGCTGAAG	2811
RDX	NM_001009576.1	F:GAATTCCGATGCCGAAACCGATCAATG<br>R:GGTACCCTCACATTGCTTCAAACTCATC	1752

Note: Italic portion indicate enzyme sites.

1.2 Primer sequences for inhibit expression level of NCOA3 were shown in Supplementary Table 2.

Supplementary Table 2. Primers and nominations of siRNA

Interfering genes	Name	Primer sequences（5’-3’）
NCOA3	siNCOA3-1	CGAGCAGUGUCUCUAGAUAGC
	siNCOA3-2	GGAGAGUAAAUAUAUUGAAGA
	siNCOA3-3	AGUUGUUAGUAUAGAUACAAA
	siNCOA3-NC	GCGAUUGAGCUGGUCACUATT

1.3 The primer sequences for detect the changes of CyclinD1 and NCOA3 were shown in Supplementary Table 3.

Supplementary Table 3. Primer sequences for the CyclinD1 and NCOA3

Gene	Gene bank	Primer sequences（5’-3’）	Production length(bp)
CyclinD1	XM_021082686.1	F: TGCATCTACACCGACAACTCCA<br>R: GTTGGAAATGAACTTCACGTCTGT	222
NCOA3	NM_001114276.1	F: GAGGGTGTGGGGACTTCTCTTT<br>R:GGGACTCTTGGAATCCTGACTG	120
GAPDH	NM_001206359.1	F: CGGCACAGTCAAGGCGGAGAAC<br>R: CATCGGCAGAAGGGGCAGAGAT	212

1.4 The effects of overexpression of NCOA3, DDR2 and RDX on cell viability of PSCs were shown in Supplementary Table 4.

Supplementary Table 4. The effects of overexpression of NCOA3, DDR2 and RDX on cell viability compared with the NC group

Group	OD_{450} in D1	OD_{450} in D2	OD_{450} in D3	OD_{450} in D4	OD_{450} in D5	OD_{450} in D6	OD_{450} in D7
pCMV-HA	0.238 ± 0.005[a]	0.277 ± 0.004[a]	0.316 ± 0.004[a]	0.488 ± 0.008[a]	0.724 ± 0.013[a]	0.946 ± 0.011[a]	1.406 ± 0.014[a]
NCOA3	0.240 ± 0.004[a]	0.285 ± 0.003[a]	0.395 ± 0.017[b]	0.625 ± 0.015[b]	0.984 ± 0.019[b]	1.267 ± 0.038[b]	1.717 ± 0.044[b]
DDR2	0.245 ± 0.004[a]	0.281 ± 0.009[a]	0.311 ± 0.008[a]	0.453 ± 0.012[a]	0.719 ± 0.012[a]	0.978 ± 0.008[a]	1.430 ± 0.013[a]
RDX	0.238 ± 0.004[a]	0.276 ± 0.007[a]	0.331 ± 0.014[a]	0.501 ± 0.009[a]	0.713 ± 0.018[a]	0.938 ± 0.010[a]	1.383 ± 0.039[a]

Note: Compared with the NC group, different letters means the difference was significant at the same time ($p<0.05$).

1.5 The effects of interfering expression of NCOA3 on cell viability of PSCs were shown in Supplementary Table 5.

Supplementary Table 5. The effects of interfering expression of NCOA3 on cell viability compared with the NC group

Group	OD_{450} in D1	OD_{450} in D2	OD_{450} in D3	OD_{450} in D4	OD_{450} in D5	OD_{450} in D6	OD_{450} in D7
siNCOA3-NC	0.225 ± 0.002[a]	0.278 ± 0.013[a]	0.342 ± 0.012[a]	0.461 ± 0.019[a]	0.730 ± 0.010[a]	0.971 ± 0.010[a]	1.361 ± 0.008[a]
siNCOA3	0.223 ± 0.003[a]	0.282 ± 0.007[a]	0.320 ± 0.010[a]	0.370 ± 0.009[b]	0.553 ± 0.017[b]	0.803 ± 0.009[b]	0.925 ± 0.011[b]

Note: Compared with the NC group, different letters means the difference was significant at the same time ($p<0.05$).

1.6 The effects of co-transfection of TA-V1 and NCOA3 on cell viability of PSCs were shown in Supplementary Table 6.

Supplementary Table 6. The effects of co-transfection of TA-V1 and NCOA3 on cell viability compared with the NC group

Group	OD_{450} in D1	OD_{450} in D2	OD_{450} in D3	OD_{450} in D4	OD_{450} in D5	OD_{450} in D6	OD_{450} in D7
pCMV-HA	0.215 ± 0.003[a]	0.266 ± 0.001[a]	0.342 ± 0.012[a]	0.477 ± 0.011[a]	0.758 ± 0.011[a]	0.965 ± 0.017[a]	1.480 ± 0.026[a]
TA-V1	0.213 ±0.002[a]	0.266 ± 0.004[a]	0.340 ± 0.015[a]	0.412 ± 0.010[b]	0.606 ± 0.010[b]	0.803 ± 0.009[b]	0.971 ± 0.011[b]
NCOA3	0.217 ± 0.002[a]	0.262 ± 0.002[a]	0.367 ± 0.003[b]	0.682 ± 0.005[b]	0.988 ± 0.001[b]	1.317 ± 0.003[b]	1.894 ± 0.002[b]
TA-V1 +NCOA3	0.217 ± 0.003[a]	0.261 ± 0.001[a]	0.333 ± 0.002[a]	0.430 ± 0.003[b]	0.667 ± 0.003[b]	0.863 ± 0.003[b]	1.367 ± 0.009[b]

Note: Compared with the NC group, different letters means the difference was significant at the same time ($p<0.05$).
